# Selbst eingeschätzte kognitive Glukosesensitivität: Zusammenhang mit Langzeitblutzuckerspiegel und diabetesbedingter Belastung bei Individuen mit Typ-1-Diabetes

**DOI:** 10.1007/s11553-023-01017-8

**Published:** 2023-02-24

**Authors:** Tobias Neukirchen, Larissa Franziska Buitkamp, Christian Vorstius

**Affiliations:** Lehrstuhl für Allgemeine und Biologische Psychologie, Bergische Universität Wuppertal, Wuppertal, Deutschland Max-Horkheimer-Str. 20, 42119

**Keywords:** Blutzucker, Psychologisches Funktionsniveau, Selbstauskunft, Glykiertes Hämoglobin, Fragebogen, Blood sugar, Psychological functioning, Self-report, Glycated hemoglobin, Questionnaire

## Abstract

**Hintergrund:**

Diabeteserkrankungen gehen häufig mit deutlichen Einschränkungen des psychischen Funktionsniveaus und Wohlergehens einher. Effektive Prävention und Gesundheitsförderung betroffener Personen setzt ein tiefgreifenderes Verständnis dieser Problematik voraus, welche der Interaktion psychischer und biologischer Prozesse entspringt.

**Ziel der Arbeit:**

Die Studie soll einen Beitrag zum Verständnis leisten, inwiefern subjektiver kognitive Glukosesensitivität (kGS) mit Langzeitblutzucker (HbA1c) und diabetesbedingter Belastung bei Personen mit Diabetes Typ 1 zusammenhängen.

**Material und Methoden:**

Im Rahmen einer Online-Studie wurden die relevanten Variablen (kGS, letzter Laborwert HbA1c, diabetesbedingte Belastung) ökonomisch mit Selbstberichtsfragebögen erfasst.

**Ergebnisse:**

In der Stichprobe aus 354 erwachsenen Personen mit Typ-1-Diabetes (283 Frauen) fanden wir signifikante Korrelationen zwischen kGS und dem HbA1c (*r*[352] = 0,133, *p* = 0,006) sowie der diabetesbedingten Belastung (*r*[352] = 0,242, *p* < 0,001). Der HbA1c korrelierte auch signifikant mit der diabetesbedingten Belastung (*r*[352] = 0,223, *p* < 0,001).

**Schlussfolgerung:**

Die Ergebnisse weisen darauf hin, dass die kGS physiologisch bedingt ist und in Zusammenhang mit etablierten, diabetesrelevanten Messgrößen (HbA1c und diabetesbedingter Belastung) steht. Insgesamt bekräftigen die gewonnen Erkenntnisse die Notwendigkeit vertiefender Erforschung und Entwicklung zugunsten einer ganzheitlichen Versorgung von Personen mit Diabetes, dessen integraler Bestandteil das Erleben und Verhalten Betroffener ist. Langfristig könnte so geklärt werden, inwiefern die Behandlung der kGS präventiv gegen die negativen Effekte von Diabeteserkrankungen auf die Psyche wirken kann und so der Gesundheitsförderung betroffener Personen zuträglich wäre.

## Abstract (en)

**Background:**

Diabetes is often associated with a significant impact on psychological functioning and well-being. Effective prevention and health promotion of persons with diabetes requires a deeper understanding of this problem, which is based on the interaction of psychological and biological processes.

**Objectives:**

The study aims to contribute to the understanding of how subjective cognitive glucose sensitivity (cGS) is related to long-term blood glucose levels (HbA1c) and diabetes-related distress in people with type 1 diabetes.

**Materials and methods:**

As part of an online study, the relevant variables (cognitive glucose sensitivity [cGS], latest measured HbA1c, diabetes-related distress) were recorded economically using self-report questionnaires.

**Results:**

In a group of 354 adults with type 1 diabetes (283 women), we found significant correlations between cGS and HbA1c (*r*[352] = 0.133, *p* = 0.006) and diabetes-related distress (*r*[352] = 0.242, *p* < 0.001). HbA1c was also significantly correlated with diabetes-related distress (*r*[352] = 0.223, *p* < 0.001).

**Conclusion:**

Our results indicate that cGS is physiologically determined and related to established diabetes-related measures (HbA1c and diabetes-related stress). Overall, the findings reaffirm a need for intensified research and development of comprehensive care for persons with diabetes, integrating experience and behaviors of the affected person’s mind as a substantial part. Ultimately, this could clarify the extent to which the treatment of cGS can have a preventive effect against negative impacts of diabetes on psychological well-being and would, thus, be beneficial to the health promotion of affected individuals.

## Einleitung

### Hintergrund

Diabetes beeinträchtigt nicht nur das körperliche, sondern auch das psychische Wohlbefinden betroffener Personen [13, 18]. Die Berücksichtigung psychischer, einschließlich kognitiver, emotionaler und verhaltensbezogener Aspekte, rückt vermehrt in den Fokus aktueller Forschung, da sie von entscheidender Bedeutung für das Patientenwohl sind [3, 6, 9, 13, 15, 18, 19, 39].

Autoren wie Kalra et al. argumentieren, dass die Vernachlässigung psychischer Aspekte eines Diabetes vorwiegend auf den Mangel an geschultem Personal, Zeit und Ressourcen zurückzuführen sei [18]. Mit Blick auf die mögliche Vermeidung psychischer Folgeerkrankungen [7, 9, 10, 24] und die – durch psychische Faktoren vermittelte [6, 7, 9, 24] – Verbesserung der Therapieadhärenz erscheinen Investitionen in diese Bereiche jedoch ausgesprochen sinnvoll.

Konstrukte wie die sog. diabetesbedingte Belastung wurden eigens zum Zweck der Operationalisierung der Gesamtheit diabetesbedingter psychischer krankheits- und therapiebedingter Faktoren entwickelt. Sie umfassen die Besorgnis des Patienten hinsichtlich des Krankheitsmanagements, sozialer Unterstützung, emotionaler Belastung sowie bezüglich des Zugangs zu medizinischer Versorgung [32]. Mit Fragebögen wie der Problem Areas in Diabetes Scale (PAID-Skala) wurde Praxis und Forschung ein einfaches Instrument an die Hand gegeben, das eben jene subjektiven Herausforderungen der Patientinnen/Patienten erfasst. Diabetesbedingte Belastung ist dabei mit entscheidenden Patientenvariablen wie Adhärenz von Bewegungsvorgaben und Medikamenten [1, 30], HbA1c-Wert und glykämischer Kontrolle [14, 30] assoziiert.

Selbst im subklinischen Bereich sind die Zusammenhänge zwischen Variablen des glukoregulativen Systems und psychischen Aspekten evident. So gehen beispielsweise bei gesunden Personen höhere Blutzuckerspiegel im Normalbereich mit geringeren Volumina der grauen/weißen Substanz in den frontalen Kortizes einher, was sich auf behavioraler Ebene wiederum in schlechteren Ergebnissen in psychologischen Leistungstests äußert [26, 34]. Das Ausmaß dieser Effekte ist dabei z. T. gravierend – Personen mit einem Blutzuckerspiegel im Prädiabetesbereich unterliefen in einer Inhibitionsaufgabe fast doppelt so viele Fehler wie Personen mit normalem Blutzuckerspiegel [12].

Mit dem Konstrukt der kognitiven Glukosesensitivität (kGS) wurde ein weiteres Instrument geschaffen, welches eine genauere Betrachtung des Wechselspiels von psychischen Prozessen und Glukosehaushalt ermöglichen soll. Es handelt sich dabei um die individuelle Responsivität mentaler Vorgänge auf die externe Zufuhr von Glukose [28]. Diese wurde bisher experimentell gemessen, indem entsprechende Leistungsparameter individuell, jeweils mit und ohne externe Glukosezufuhr, erhoben wurden. Während eine niedrige kGS für eine geringe Veränderung psychischer Parameter infolge der Glukosezufuhr steht, ist eine hohe kGS als eine starke, glukosebedingte Verbesserung dieser Parameter zu verstehen. Die im Experiment festgestellten Effekte der kGS sind dabei erheblich – Personen mit den schlechtesten Leistungen im Fastenzustand konnten ihre Leistung unter Glukosezufuhr teilweise mehr als verdoppeln [28]. Bei den Personen mit den besten Leistungen im Fastenzustand konnte in derselben Studie hingegen keine große kGS nachgewiesen werden. Die kGS stellt also ein individuelles und situatives Leistungsdefizit dar, welches durch eine Glukosezufuhr kurzfristig teilkompensiert werden kann. Neuheitswert besitzen dabei v. a. zwei Aspekte. Erstens, die starke Vorhersagekraft der Leistung in Abwesenheit einer Glukosezufuhr auf die zu erwartende Leistungssteigerung durch eine Glukosezufuhr. Wenn eine Person ohne kurzfristig vorangegangene Glukosezufuhr schlechte kognitive Leistung erbringt, dann ist die Wahrscheinlichkeit groß, dass eine Glukosezufuhr die Leistung verbessern wird. Ein solcher Effekt, insbesondere dieser Größenordnung, ist alles andere als intuitiv, wenn man die Vielzahl an – oftmals dispositionalen und zeitlich stabilen – Determinanten interindividueller Unterschiede kognitiver Leistungen berücksichtigt. Der zweite Aspekt mit Neuheitswert liegt darin, dass es sich infolge einer Glukosegabe lediglich auf intraindividueller Ebene um eine Leistungszunahme handelte. Interindividuell betrachtet blieb in derselben Studie das Leistungsniveau von Personen mit hoher kGS stets unter dem von Personen mit niedriger kGS, die sowohl fastend als auch unter Glukosezufuhr die besten Leistungen erbrachten.

Die Größe des beschriebenen Effekts spricht für eine deutliche praktische Signifikanz der kGS. Eine Leistungsveränderung, die > 50 % entsprach, fand sich in einem Drittel aller gesunden Versuchspersonen – dasselbe Drittel, dass auch das letzte Drittel der Ränge der kognitiven Leistungstests belegte [28]. Beachtet man, dass die Stichprobe verhältnismäßig jung war (M = 23,17; SD = 6,75), so ist eine praktische Relevanz eines solches Effekts im klinischen als auch im pädagogischen Kontext sowie im größeren Rahmen der Gesundheitsförderung denkbar.

Die durch die kGS quantifizierten individuellen Unterschiede in der kognitiven Sensitivität für die Einflüsse von Glukose stehen im Einklang mit Ergebnissen hinsichtlich der unter kognitiver Last verstoffwechselten Glukosemenge (gemessen über die Atemluft; [29]), weshalb der individuelle Glukosehaushalt als mediierende Größe sehr plausibel erscheint.

Angesichts der Tatsache, dass die kGS bereits bei klinisch unauffälligen Personen erhebliche interindividuelle Varianz aufweist [28], ist die Untersuchung der kGS bei Personen mit pathologisch verändertem Glukosehaushalt besonders interessant. Sowohl der Anwendung als auch der Forschung kommt dabei zugute, dass das Konstrukt der kGS nicht zwangsläufig mit dem relativ aufwendigen Verfahren – bestehend aus zwei Laborsitzungen mit und ohne externe Glukosezufuhr – erfasst werden muss. Ersten Erkenntnissen zufolge sind die Auswirkungen der kGS profund genug, um von den Betroffenen wahrgenommen und bewusst beschrieben werden zu können [27]. Grundsätzlich sind durch den Glukosehaushalt bedingte psychische Effekte durchaus introspektiv zugänglich, wie es sowohl in der Praxis durch etablierte Fragebögen zur diabetesbedingten Belastung oder auch durch zahlreiche Publikationen belegt ist [5, 42]. Auch unter Berücksichtigung der zuvor beschriebenen individuellen glukosebedingten Leistungsunterschiede von bis zu > 200 % ist es nicht sonderlich überraschend, dass Betroffene die kGS selbst an sich wahrnehmen können.

Somit liegt es nahe, dass Einschränkungen des psychischen Funktionsniveaus, wenn kürzlich keine Glukosequellen konsumiert wurden, introspektiv operationalisierbar sind. Diesen Umstand macht sich der Fragebogen *Indicators of Glucose-Dependency *(IGlu) zu Nutze, der das Ausmaß oben beschriebener Effekte erfasst und somit die *subjektive* kGS misst. Mit der subjektiven kGS wird also der Grad der selbst wahrgenommenen Einschränkungen des psychischen Erlebens – insbesondere der kognitiven Verarbeitung – beschrieben, welche mit zunehmender Nahrungsdeprivation auftritt.

Diese Größe kovariiert mit ihrem experimentell gemessenen Gegenstück und beide lassen sich durch Interventionen, welche mit Gewichtsreduktionen einhergingen, positiv beeinflussen [27]. Diese Erkenntnis stimmt insofern optimistisch, als dass die kGS insbesondere bei Männern mit dem Körpergewicht assoziiert ist [28]. Angesichts des etablierten Zusammenhangs zwischen Körpergewicht und Insulinsensitivität sowie der Evidenz hinsichtlich der moderierenden Rolle des Geschlechts [25, 37, 41] gelten Insulinsensitivität und Glukosehaushalt als die wahrscheinlichsten Mechanismen hinter der kGS [28]. Die Erforschung der kGS bei Personen mit pathologischen Auffälligkeiten des Glukosehaushalts ist somit ein naheliegender nächster Schritt, welcher im Rahmen der hier vorgestellten Studie begangen wurde. Dabei bot sich zunächst die nähere Betrachtung der subjektiven kGS an, welche die kontaktfreie, online-gestützte Untersuchung von Menschen mit Diabetes – einer Risikogruppe für COVID-19 („coronavirus disease 2019“; [38]) – während der Pandemie ermöglichte.

Das erste wesentliche Ziel der Studie ist die Untersuchung eines möglichen Zusammenhangs zwischen subjektiver kGS und einem etablierten Indikator des Langzeitblutzuckerspiegels, dem HbA1c-Wert. Ein solcher Zusammenhang wäre insofern bedeutsam, als dass er einen weiteren Hinweis für die enge Beziehung zwischen Blutglukoseregulation und psychischen Prozessen darstellen würde, welche ultimativ das Wohlbefinden des Patienten beeinflussen. Wenn das Patientenwohl priorisiert werden soll, müssen es ebenso jene Mechanismen, welche den Einfluss zwischen Soma und Psyche vermitteln. Der Grund dafür ist, dass eine hohe kGS per Definition ein situatives Leistungsdefizit darstellt, deren physiologischen Mechanismen wir im Glukosestoffwechsel und den mit ihm relatierten Faktoren vermuten. Beim Langzeitblutzuckerwert und der diabetesbedingten Belastung handelt es sich um eben solche. Daher erwarten wir jeweils positive, korrelative Zusammenhänge zwischen subjektiver kGS und den beiden unmittelbar diabetesrelatierten Konstrukten (diabetesbedingter Belastung und HbA1c-Wert). Die Annahme ist dabei, dass je schlechter die Glukoregulation ist (hoher HbA1c), desto störanfälliger ist das psychische Funktionsniveau, wenn keine externen Glukosequellen zugeführt werden. Ein solches Ergebnis stünde im Einklang mit etablierten Befunden hinsichtlich zerebraler Insulinsensitivität, individueller Anpassung an die Verwertung von Ketonkörpern sowie dem Zusammenhang zwischen Leistungsdefiziten und erhöhtem Glukoseumsatz unter kognitiver Belastung [17, 20, 29, 31].

Zweitens sollte untersucht werden, inwiefern eine Beziehung zwischen subjektiver kGS und diabetesbedingter Belastung besteht. Da die subjektive kGS nach dem aktuellen Forschungsstand beeinflussbar ist, würde ein Zusammenhang zwischen den beiden Konstrukten einen Ansatz zur Reduktion der diabetesbedingten Belastung durch Senkung der kGS darstellen. Im Gegensatz zur diabetesbedingten Belastung ist die kGS ein psychologisches Konstrukt, welches sich auch bei Personen ohne Diabetes einsetzen lässt. Die Erforschung der Beziehung zwischen beiden Konstrukten birgt somit langfristig auch das Potenzial, diabetesspezifische von diabetesunspezifischen Einflüssen auf das Erleben und Verhalten zu differenzieren oder auch psychische Effekte des Glukosehaushalts über das gesamte Kontinuum der Insulinresistenz [23] zu erforschen.

## Methode

Alle Methoden standen im Einklang mit Richtlinien der Ethikkommission der Universität (Gutachtennummer MS/BBL 210329 Neukirchen Buitkamp) und der aktuellen Fassung der Deklaration von Helsinki. Zu Beginn der Datenerhebung wurde von allen Teilnehmenden das Einverständnis zur Teilnahme und Datenverarbeitung eingeholt.

### Stichprobe

Die Rekrutierung der Probanden erfolgte über soziale Medien sowie über Aushänge in diabetologischen Schwerpunktpraxen. Die Erhebung der Daten fand zwischen April und Juni 2021 statt. Die Ad-hoc-Stichprobe bestand nach Datenbereinigung (s. unten) aus *n* = 354 erwachsenen Personen mit Typ-1-Diabetes (80 % weiblich) im Alter von 18 bis 72 Jahren (*M* = 37,30; *SD* = 12,32). Die Körpergröße lag zwischen 150 und 198 cm (*M* = 170,35; *SD* = 8,25) und das Gewicht betrug zwischen 43 und 150 kg (*M* = 79,30; *SD* = 19,40). Der BMI lag damit im Mittel bei *M* = 27,27 (*SD* = 6,21). Eine A‑priori-Poweranalyse, die auf der konservativen Erwartung kleiner Effektstärken beruhte [4, 11], ergab eine minimale Stichprobengröße von 320 Personen für eine angestrebte Power von 0,95 bei einem α = 0,05.

### Fragebögen

#### Demografischer Fragebogen

Der demografische Fragebogen erfasste anthropometrische und demografische Daten. Des Weiteren wurden Informationen bezüglich der Diabeteserkrankung erfragt, wobei in der hier vorgestellten Studie dem aktuellsten HbA1c-Wert (mit dazugehörigem Bestimmungsdatum) die größte Bedeutung zukommt. Der HbA1c-Wert wird als Indikator der durchschnittlichen Blutzuckerkonzentration verwendet. Er gibt Auskunft über die Bindung von Zucker an Hämoglobin über einen Zeitraum von 6 bis 8 Wochen [21]. Dabei bewegt sich ein HbA1c zwischen 4–6 % laut der International Federation of Clincal Chemistry (IFCC) und dem National Glycohemoglobin Standardization Program (NGSP) im Normbereich [8]. Laut der American Diabetes Association gilt als grobes Therapieziel die Aufrechterhaltung eines HbA1c-Wertes von < 7 % [2]. Ist der HbA1c-Wert über einen längeren Zeitraum erhöht, so steigt das Risiko für Folgeerkrankungen [36]. In Deutschland wird bei Diabetikern die Bestimmung des HbA1c einmal pro Quartal empfohlen, da er Rückschlüsse über den Erfolg oder Misserfolg der Diabetestherapie und somit das Risiko für Folgeerkrankungen aufzeigt [2, 8]. Diesen Umstand machten wir uns in der vorliegenden Studie zu Nutze.

#### IGlu

Die oben eingeführte subjektive kGS wurde mit dem Selbstberichtsfragebogen „indicators of glucose-dependency“ (IGlu) erfasst [27]. Hierbei handelt es sich um einen Selbstberichtsfragebogen mit 38 Items. Die Items enthalten Zustimmungen zu Aussagen über die subjektiv empfundene Abhängigkeit des emotionalen, kognitiven und physischen Funktionsniveaus von einer unmittelbaren Nahrungsaufnahme. Dabei liegt der Fokus auf den subjektiven Verschlechterungen besagter innerer Zustände und Prozesse, wie beispielsweise Unachtsamkeit, Vergesslichkeit und Gereiztheit in Abhängigkeit von der letzten Nahrungsaufnahme.

Der IGlu-Fragebogen erfasst das Ausmaß besagter Effekte über Items, welche sich auf Einschränkungen in gedächtnisbezogenen und problemlösenden kognitiven Prozessen, der Wahrnehmung und Regulation von Emotionen sowie der Wahrnehmung des eigenen Körpers beziehen. Ergänzt werden diese Aspekte um selbstsuggestive Überzeugungen, welche Einschränkungen der Leistungsfähigkeit in der Abwesenheit einer unmittelbaren Nahrungsaufnahme begünstigen könnten sowie Items zur Erfassung potenziell relevanter Gesundheitsverhaltensweisen und Lebensumstände. Aufgrund der Art ihrer Operationalisierung mittels Fragebogen wird der individuelle Gesamttestwert auch als *subjektive *kGS bezeichnet. Der Gesamttestwert ist das arithmetische Mittel aller Items, die mithilfe eines Zustimmungsreglers mit einem Wert zwischen 0 (vollkommende Ablehnung) und 100 (vollkommende Zustimmung) bewertet werden, so dass dieser auch einen Wert zwischen 0 und 100 annimmt.

#### PAID

Der Selbstberichtsfragebogen „problem areas in diabetes“ (PAID) von Polonsky et al. aus dem Jahr 1995 erfasst die diabetesbedingte Belastung. Das Messinstrument umfasst 20 Items, welche vier Subskalen (emotionale Probleme, therapiebezogene Probleme, ernährungsbezogene Probleme und Probleme, die die soziale Unterstützung betreffen) zugeordnet sind. Aus den Subskalenwerten kann ein Gesamttestwert aggregiert werden, welcher in der vorliegenden Studie zur Hypothesenprüfung herangezogen wurde. Der PAID-Testscore wurde gemäß den Vorgaben von Polonsky et al. [32] berechnet, indem die einzelnen Rohwerte aufsummiert und dann mit dem Faktor 1,25 multipliziert wurden, so dass sich Werte zwischen 0 und 100 ergeben. Die interne Konsistenz des Instruments ist sowohl im englischsprachigen Original (α = 0,95) als auch in der deutschen Version (α = 0,93) belegt [32].

### Datenaufbereitung und statistische Analyse

Die Daten wurden in R ausgewertet [33]. Insgesamt begannen *n* = 812 Personen mit der Bearbeitung der Fragebögen. Nachdem die Daten auf Stichprobenebene bereinigt wurden (Einwilligung der anonymisierten Datenerfassung erteilt, vollständige Bearbeitung aller Fragen, HbA1c > 0 und < 20, Dauer der Erkrankung < Lebensalter) verblieben 416 Versuchspersonen. Ausgehend von der mittleren Lebensdauer menschlicher Erythrozyten [40] wurden lediglich Fälle zur Analyse zugelassen, bei denen der HbA1c vor weniger als 121 Tagen vor Teilnahme an der Studie ermittelt wurde. Es verblieben damit *n* = 354 Personen zur Analyse in der Stichprobe. Das Signifikanzniveau für alle statistischen Tests wurde auf α = 0,05 festgelegt.

### Ablauf

Die Datenerhebung erfolgte Online und anonymisiert über die Plattform SoSci Survey [22]. Nachdem die Versuchspersonen den Datenschutzerklärungen zugestimmt hatten, wurde ihnen der demografische Fragebogen vorgelegt. Im Anschluss wurde die diabetesbedingte Belastung mithilfe des Fragebogens „Problem Areas in Diabetes“ [32] und die subjektive kGS mit dem IGlu-Fragebogen [27] erfasst.

## Ergebnisse

In Tab. 1 findet sich eine Übersicht der Ergebnisse der deskriptiven Analyse der physischen und hypothesenrelevanten Variablen aller Teilnehmenden, deren Datensätze den Selektionskriterien (vgl. Datenaufbereitung und statistische Analyse oben) entsprachen.

**Tab. 1:** Deskriptive Statistiken zu erhobenen Variablen innerhalb der untersuchten Stichprobe

	*M*	*SD*	*Range*	*Min*	*Max*
Lebensalter	37,299	12,319	54	18	72
Jahre seit Diagnose	15,952	12,522	54	0	54
Körpergewicht	79,297	19,404	107,000	43,000	150,000
Körpergröße	170,345	8,248	48	150	198
BMI	27,267	6,209	35,207	15,605	50,811
HbA1c	7,094	1,641	12,000	4,900	16,900
Aktualität HbA1c	45,674	30,763	119,956	0	120,383
PAID-Testwert	58,129	20,734	91,250	25,000	116,250
IGlu-Testwert	44,831	11,869	66,868	15,684	82,553

Lebensalter in Jahren, Körpergewicht in kg, Körpergröße in cm, HbA1c in %, Aktualität des HbA1c in Tagen (Differenz Teilnahmedatum – Datum der angegebenen HbA1c-Messung), *N* = 354

*BMI* Body Mass Index, *M* Mittelwert, *SD* Standardabweichung, *PAID* „problem areas in diabetes“, *IGlu* „indicators of glucose-dependency“, *Min* Minimum, *Max* Maximum, *HbA1c* Langzeitblutzucker

Die Korrelation zwischen IGlu und HbA1c erreichte statistische Signifikanz mit *r*(352) = 0,133, *p* = 0,006. Auch Prüfung des angenommenen Zusammenhangs zwischen den Testwerten des IGlu und PAID ergab eine hochsignifikante Korrelation mit *r*(352) = 0,242, *p* < 0,001. Zusätzlich wurde ein möglicher Zusammenhang zwischen HbA1c und PAID untersucht, welcher sich ebenfalls als statistisch hochsignifikant erwies mit *r*(352) = 0,223, *p* < 0,001. Vertiefend wurde geprüft, inwiefern der Zusammenhang zwischen IGlu und HbA1c durch die diabetesbedingte Belastung bedingt sein könnte. Eine entsprechende partielle Korrelation zwischen IGlu und HbA1c, bei der der Einfluss der diabetesbedingten Belastung kontrolliert wurde, ergab eine tendenziell signifikante Assoziation zwischen IGlu und HbA1c: *r*(352) = 0,084, *p* = 0,058. Die beschriebenen Zusammenhänge blieben in der gleichen Stärke auch erhalten, wenn in den Analysen für Alter kontrolliert wurde.

In Zusatzanalysen wurden mögliche geschlechtsspezifische Effekte untersucht. Dazu wurden die Zusammenhänge von IGlu, HbA1c und PAID getrenntgeschlechtlich betrachtet für zwei der drei überprüften Zusammenhänge interessante Unterschiede zwischen den Geschlechtern entdeckt. Während der Zusammenhang zwischen IGlu und HbA1c für Männer und Frauen vergleichbar war (*r*[281] = 0,138, *p* = 0,020; *r*[69] = 0,124, *p* = 0,303), zeigte sich für den Zusammenhang zwischen IGlu und PAID eine substantiell höhere Korrelation in der männlichen Substichprobe (*r*[69] = 0,517, *p* < 0,001) im Vergleich zur Weiblichen (*r*[281] = 0,170, *p* = 0,004). Auch für den Zusammenhang zwischen HbA1c und PAID fand sich eine wesentlich ausgeprägtere Korrelation für die männliche (*r*[69] = 0,409, *p* < 0,001) als die weibliche Substichprobe (*r*[281] = 0,180, *p* = 0,002). Analog zur Gesamtstichprobe, fand sich in der weiblichen Substichprobe ebenfalls eine wesentliche Reduktion des Zusammenhangs zwischen IGlu und HbA1c, wenn für PAID kontrolliert wurde (*r*[281] = 0,084, *p* = 0,117).

## Diskussion

In der vorliegenden Studie überprüften wir mögliche Zusammenhänge zwischen der subjektive kGS und dem Langzeitblutzuckerspiegel sowie der diabetesbedingten Belastung in einer Stichprobe aus Personen mit Typ-1-Diabetes.

Weiterhin untersuchten wir innerhalb derselben Stichprobe die Beziehung zwischen diabetesbedingter Belastung und dem HbA1c.

Unsere Ergebnisse stützen die Hypothesen und zeigen Zusammenhänge zwischen den genannten Konstrukten und untermauern damit unsere ursprüngliche Annahme, dass eine höhere Störanfälligkeit des psychischen Funktionsniveaus unter Nahrungsdeprivation (skGS) mit einer schlechteren Glukoregulation (hoher HbA1c) einhergeht. Die Beziehung zwischen skGS und diabetesbedingter Belastung ist ein Indiz der Relevanz kognitiver Einschränkungen für die Folgen einer Diabeteserkrankung für das psychische Wohlbefinden.

Im Einzelnen gelang bei dieser erstmaligen Anwendung des IGlu-Fragebogens zur Messung der subjektiven kGS in einer klinisch relevanten Stichprobe der Nachweis einer Assoziation mit dem HbA1c. Die subjektive kGS stand zudem mit der diabetesbedingten Belastung im Zusammenhang. Darüber hinaus erfolgte die Replikation der etablierten Beziehung zwischen diabetesbedingter Belastung und HbA1c [4, 11]. Diese Ergebnisse stehen im Einklang mit der Annahme, dass das Konstrukt der kGS physiologisch bedingt ist [28, 29] und sind damit für dessen weitere Erforschung richtungsweisend.

Die Erkenntnis, dass die diabetesbedingte Belastung wesentlich zu der Beziehung zwischen kGS und HbA1c beiträgt, ist in mehrerlei Hinsicht interessant. Die subjektive Eigenwahrnehmung der kGS bei Personen mit Typ-1-Diabetes scheint substantiell durch die emotionalen Folgen der Diabeteserkrankung beeinflusst zu werden. Dies hat zweierlei mögliche Implikationen. Einerseits könnte die Introspektion durch die Belastung negativ beeinflusst sein – Betroffene starker diabetesbedingter Belastung schätzen die Abhängigkeit ihrer kognitiven Fähigkeiten von externen Glukosequellen als größer ein als diese tatsächlich ist. Andererseits wäre es denkbar, dass die diabetesbedingte Belastung tatsächlich Auswirkungen auf die kGS hat und betroffene Personen vulnerabler für die kognitiven Folgen von Nahrungsdeprivation macht.

Darüber hinaus stellt sich die Frage, durch welche Konstrukte eine mögliche Assoziation zwischen HbA1c und kGS bei Personen ohne Diabetes vermittelt werden würde. Angesichts der bereits etablierten Mediatorrolle diabetesbedingter Belastung zwischen HbA1c und depressiver Symptomatik [4] ist auch ein Effekt der kGS bei der Entwicklung depressiver Symptome denkbar. Die Assoziation depressiver Symptomatik mit Einschränkungen der kognitiven Verarbeitung emotionaler Stimuli gilt als etabliert [16, 35]. Unter Berücksichtigung der hohen Vulnerabilität von Personen mit Diabetes [3, 14, 15] ist es naheliegend, zukünftig auch spezielle Einschränkungen des kognitiven Funktionsniveaus – wie die kGS – im Kontext von Diabetes und Depressivität zu untersuchen.

Die etablierten Korrelate der diabetesbedingten Belastung, wie Adhärenz von Bewegungsvorgaben und Medikamenten [1, 30] und glykämische Kontrolle [14, 30], könnten ihrerseits den Zusammenhang mit der kGS beeinflussen. Beispielsweise könnten Variablen wie die Adhärenz protektive Faktoren darstellen, die determinieren, ob ein erhöhter HbA1c tatsächlich zu den situativen Einschränkungen des psychischen Funktionsniveaus führt, welche die kGS konstituieren. Entsprechend elaborierte, konsekutive Studien, welche sowohl über Methoden zur Erfassung weitere Konstrukte als auch angepasste Untersuchungsdesigns verfügen, könnten wesentlich zur Beantwortung dieser Fragen beitragen. Dabei gilt es, sowohl die wissenschaftlichen Erkenntnisse als auch die identifizierten Limitationen der vorgestellten Studie aufzugreifen, wie z. B. das auf Selbstauskunft des HbA1c basierende Design. Dies stellt einen Kompromiss dar, den bereits wesentliche Pionierarbeiten des Feldes eingehen mussten [11] und den es in zukünftigen Projekten zu überwinden gilt. Eine mögliche Lösung könnte durch die Verwendung von Krankenakten der Patienten erzielt werden, z. B. durch die unmittelbare Durchführung der Studie in Zusammenarbeit mit klinischen Einrichtungen und Behandlungszentren. Diesbezüglich sollten zukünftige Untersuchungen ebenfalls überprüfen, ob und inwiefern sich ähnliche Resultate bei Personen mit Typ-2-Diabetes replizieren lassen. Dabei ist zu beachten, dass viele Personen mit Typ-2-Diabetes älter als der Bevölkerungsdurchschnitt sind [16] und somit die Rekrutierung einer Stichprobe ausreichenden Umfangs im Rahmen einer internetbasierten Umfrage eine Herausforderung sein könnte.

Die dargestellten Daten machen deutlich, dass die Verwendung psychologischer Messgrößen, einschließlich der kognitiven Glukosesensitivität, wertvolle Hinweise und Erkenntnisse über mentale Prozesse darstellen. Diese können zu einem besseren Verständnis der Verbindung von physiologischen Leiden und der Qualität des Erlebens und Verhaltens betroffener Personen beitragen und perspektivisch sinnvolle Ergänzungen in der Diabetestherapie bieten. Aus diagnostischer Sicht ist dabei die Tatsache interessant, dass mit dem IGlu-Fragebogen in erster Linie kognitive Einschränkungen operationalisiert werden, während PAID überwiegend emotionale Aspekte des Erlebens erfasst. Somit ergibt sich in kombinierter Nutzung die Möglichkeit zur umfangreicheren Ermittlung des psychischen Funktionsniveaus unter Berücksichtigung der Einschränkungen im Glukosestoffwechsel. Die Involvierung höherer kognitiver Prozesse in der Konstituierung der selbsteingeschätzten kGS birgt Potenzial zur Interventionsbildung durch eine kognitive Verhaltenstherapie. Erfolgen könnte dies beispielsweise durch die Bewusstmachung von Kognitionen, welche die subjektiven Einschränkungen des eigenen Funktionsniveaus im Zusammenhang mit Glukose betreffen. Die anschließende Prüfung der Angemessenheit solcher Kognitionen könnte sowohl zur Verringerung von selbstsuggestiven Effekten als auch zur Identifikation von Symptomen suboptimaler Medikation oder Therapieadhärenz beitragen. Somit könnten entsprechende Denkmuster direkt modifiziert und negativen Effekten von Diabeteserkrankungen auf die Psyche entgegengewirkt werden. Dass sich die kGS bereits in Personen ohne Diabeteserkrankungen als valides psychologisches Konstrukt erwies, weckt Hoffnungen auf einen Mehrwert im Bereich der Prävention psychischer Negativfolgen, lange bevor die Einschränkungen des Glukosehaushalts den subklinischen Bereich verlassen.

## Limitationen

Aufgrund des verwendeten Untersuchungsdesigns lassen sich keine eindeutigen Kausalitäten aus den festgestellten Zusammenhängen ableiten. Denkbar wären transaktionale Zusammenhänge zwischen den psychischen Konstrukten und dem HbA1c, welche durch behaviorale Aspekte (z. B. niedrigere Adhärenz, emotionales Essen) vermittelt werden könnten. Weiterhin basieren die ausgewerteten Daten auf anonymen Selbstberichten von freiwilligen, interessierten Personen mit Internetzugang, weshalb introspektive Fähigkeiten, Validität der Angabe des letzten HbA1c und die Teilnahmebereitschaft als potenziell bei der Ergebnisinterpretation berücksichtigt werden sollten. Insbesondere die Geschlechterverteilung der Ad-hoc-Stichprobe entspricht nicht der Gesamtpopulation und birgt – nebst der Tatsache, dass weniger als die Hälfte aller teilnehmenden Personen den Fragebogen vollständig bearbeiteten – Verbesserungspotential für zukünftige Untersuchungen.

## Schlussfolgerung

Die Beziehung zwischen den Testwerten der beiden psychometrischen Instrumente sowie deren Assoziation mit dem HbA1c bekräftigten, dass psychische Auswirkungen einer Diabeteserkrankung vielfältig, hoch-prävalent und ökonomisch nachweisbar sind. Erwähnenswert ist dabei, dass die Korrelation zwischen kGS und diabetesbedingter Belastung lediglich von moderater Größe ist. Das spricht dafür, dass beide psychometrischen Verfahren tatsächlich unterschiedliche Konstrukte operationalisieren, wenngleich bei vorliegender Diabeteserkrankung die diabetesbedingte Belastung wesentlich zur subjektiven Bewertung der eigenen kGS beiträgt.

Weiterhin liefern die gewonnenen Ergebnisse zusätzliche Evidenz für geschlechtsspezifische Unterschiede im Kontext der kGS. Die in der Einleitung erwähnte Interaktion von Geschlecht und Körpergewicht auf die objektiv gemessene kGS [28] wirft im Zusammenspiel mit den hier vorgestellten Befunden die zukünftig zu beantwortende Frage auf, ob die kGS bei Männern allgemein stärker mit komorbiden Begleiterscheinungen (nebst höherer diabetesbedingter Belastung) einhergeht.

## Fazit für die Praxis

- Für die Praxis bedeuten unsere Ergebnisse, dass die Verwendung von psychometrischen Instrumenten, wie dem PAID („problem areas in diabetes“) oder IGlu („indicators of glucose-dependency“), lohnenswerte Optionen darstellen, um das Patientenwohl bei vorliegendem Diabetes standardisiert und differenziert zu erfassen.
- Entsprechende Instrumente können bei der Erkennung von mit der Erkrankung assoziierten Einschränkungen des psychischen Erlebens helfen und zur konkreten und zeitökonomischen Evaluation eines möglichen Handlungsbedarfs von ärztlicher/therapeutischer Seite beitragen.
- Die – insbesondere bei männlichen Patienten – ausgeprägte Assoziation zwischen HbA1c (Langzeitblutzucker) und den hier erforschten psychologischen Konstrukten sollte bei der Anamnese im diabetologischen als auch im psychiatrischen Bereich berücksichtigt werden.
